# Gastrointestinal infections caused by consumption of raw drinking milk in England & Wales, 1992–2017

**DOI:** 10.1017/S095026881900164X

**Published:** 2019-09-27

**Authors:** N. Adams, L. Byrne, J. Edge, A. Hoban, C. Jenkins, L. Larkin

**Affiliations:** 1 National Infection Service, Public Health England, UK; 2 NIHR Health Protection Research Unit in Gastrointestinal Infections, UK; 3 Food Standards Agency, London, UK

**Keywords:** Epidemiology, food safety, food-borne zoonoses, gastrointestinal infections, public health emerging infections

## Abstract

Systematic, national surveillance of outbreaks of intestinal infectious disease has been undertaken by Public Health England (PHE) since 1992. Between 1992 and 2002, there were 19 outbreaks linked to raw drinking milk (RDM) or products made using raw milk, involving 229 people; 36 of these were hospitalised. There followed an eleven-year period (2003–2013) where no outbreaks linked to RDM were reported. However, since 2014 seven outbreaks of *Escherichia coli* O157:H7 (*n* = 3) or *Campylobacter jejuni* (*n* = 4) caused by contaminated RDM were investigated and reported. Between 2014 and 2017, there were 114 cases, five reported hospitalisations and one death. The data presented within this review indicated that the risk of RDM has increased since 2014. Despite the labelling requirements and recommendations that children should not consume RDM, almost a third of outbreak cases were children. In addition, there has been an increase in consumer popularity and in registered RDM producers in the UK. The Food Standards Agency (FSA) continue to provide advice on RDM to consumers and have recently made additional recommendations to enhance existing controls around registration and hygiene of RDM producers.

## Short report

Raw drinking milk (RDM) has a diverse microbial flora which can include pathogens transmissible to humans. In the UK, RDM is most commonly sourced from cows, and to a lesser extent from goats, sheep and buffalo (https://acmsf.food.gov.uk/sites/default/files/acm_1269_revised_final.pdf). Pathogens most commonly associated with human illness following the consumption of RDM are *Campylobacter* spp., *Salmonella* spp., *Brucella melitensis*, *Mycobacterium bovis,* tick-borne encephalitis virus and Shiga Toxin-producing *Escherichia coli* (STEC) [1]. Contamination can arise from direct excretion into the milk from animals with systemic infection as well as from localised infections, such as mastitis, and faecal contamination during milking or from the wider farm environment [1].

In England and Wales, RDM can currently only be sold by registered RDM producers directly to the customer at the farm gate or farmhouse catering operation, by farmers at farmers' markets, distributors using a vehicle as a shop such as a milk round, direct online sales or vending machines at farms. In England and Wales, RDM must be labelled with a health warning. In England, this includes the statement ‘This milk has not been heat treated and may contain organisms harmful to health’, in Wales this is expanded to include ‘The Food Standards Agency strongly advises that it should not be consumed by children, pregnant women, older people or those who are unwell or have a chronic illness’. In Scotland, the sale of RDM is banned.

Systematic, national surveillance of general outbreaks of intestinal infectious disease (IID) in England and Wales has been undertaken by Public Health England (PHE) since 1992 [2]. Upon notification of an outbreak, a standardised surveillance form is sent to the consultant in communicable disease control leading the investigation with a request that it is completed once the outbreak investigation has ended. Since 2004, following an EU direction in 2003 (Directive 2003/99/EC), reporting of outbreaks of IID to PHE's electronic foodborne and non-foodborne gastrointestinal outbreak surveillance system (eFOSS) has been mandated.

Reported data on foodborne IID outbreaks are valuable for analysing links between foodborne illness and specific food vehicles or situations that cause them, monitoring trends and assessing risk. While the number of cases linked to confirmed and putative vehicles during outbreak investigations do not portray the true burden of disease, they can be useful in examining trends which may be indicative of risk exposures amongst apparently sporadic cases [3]. These data can, therefore, contribute to risk assessments and inform policy. Information from eFOSS is routinely provided to UK government departments, including the Food Standards Agency, the Department for Environmental, Food and Rural Affairs and the Department of Health; and to European agencies, specifically the European Food Safety Authority (EFSA) and European Centre for Disease Prevention and Control (ECDC).

Previous reviews of foodborne outbreaks in England and Wales between 1992 and 2008 described an overall decline in the number of foodborne outbreaks detected [4]. Despite this overall decline in foodborne outbreaks, studies published in 2003 and 2005 [5, 6] highlighted the emergence of STEC O157:H7 as a milk borne pathogen, and the role of RDM in causing outbreaks of human disease in England and Wales. A subsequent review of foodborne outbreaks in 2011 [3] described a decrease in the number of outbreaks caused by the consumption of milk and milk products, including those made from raw milk, during the study period. In that study, most milk borne outbreaks were caused by STEC O157:H7 followed by *Salmonella* and *Campylobacter* species [4]. However, a decline in the relative role of RDM as a causative agent of outbreaks of STEC O157:H7 was reported between 2002 and 2012 [3].

The aim of this short report is to provide an update on the previously published data on outbreaks and incidents involving reported human illness associated with RDM [4, 5, 6]. In this report, we use the term RDM to include RDM and dairy products containing RDM, such as cheese and cream. Here, we review all the outbreaks and incidents involving reported human illness associated with RDM, between 1992 and 2017 in England and Wales. In addition, we describe available surveillance data on sporadic STEC infections and sporadic cases of listeriosis in relation to exposure to RDM.

Between 1992 and 2001, there were 19 outbreaks (1.7 per year) linked to RDM involving 229 people (20.8 cases per year); 36 of these were hospitalised as a result of the infection (Table 1). At least one outbreak occurred every year, except in the years 1999 and 2001. The highest number of outbreaks recorded in one year was three, as observed in the years 1993, 1994, 1996 and 2000. The number of cases declined from 72 in 1992 to nine in 2002 (Table 1). No outbreaks associated with RDM were identified between 2003 and 2013.

**Table 1.** Reported foodborne IID outbreaks and IID outbreaks associated with RDM or products made using raw milk, in England and Wales, 1992–2017

Year	Total number of foodborne IID outbreaks	Number affected (number hospitalised)	Number of deaths	Total number of IID outbreaks linked to RDM	RDM outbreaks as a % of total number of foodborne outbreaks	Number affected (number hospitalised)
1992	238	6663 (237)	8	1	0.4	72 (0)
1993	249	6032 (181)	8	3	1.2	41 (9)
1994	245	5666 (186)	5	3	1.2	38 (1)
1995	236	6321 (198)	11	1	0.4	26 (7)
1996	209	4673 (210)	22	3	1.4	16 (4)
1997	254	5208 (204)	15	1	0.4	8 (2)
1998	151	3564 (94)	6	2	1.3	10 (4)
1999	123	2920 (136)	2	0	0.0	0
2000	125	3261 (101)	2	3	2.4	9 (3)
2001	105	1806 (94)	4	0	0.0	0
2002	89	2364 (78)	20	2	2.2	9 (6)
2003	83	2430 (114)	2	0	0.0	0
2004	70	1798 (71)	6	0	0.0	0
2005	87	1957 (85)	4	0	0.0	0
2006	73	1933 (76)	7	0	0.0	0
2007	52	1102 (84)	11	0	0.0	0
2008	41	919 (46)	8	0	0.0	0
2009	91	3408 (108)	8	0	0.0	0
2010	63	1418 (83)	5	0	0.0	0
2011	83	2133 (83)	3	0	0.0	0
2012	55	1324 (74)	8	0	0.0	0
2013	77	2552 (62)	13	0	0.0	0
2014	70	2055 (78)	4	1	1.4	9 (2)
2015	48	1098 (34)	1	0	0.0	0
2016	44	2629 (93)	2	2	4.5	70
2017	27	667 (24)	0	4	14.8	34 (3)
Total	2988	75 901 (2834)	185	26	0.9	357 (41)

More recently between 2014 and 2017, seven outbreaks (1.75 per year) caused by RDM have been investigated and reported, involving 114 people (28.5 cases per year), five reported hospitalisations and one death (Table 2). The seven outbreaks were caused by *E. coli* O157:H7 (*n* = 3) or *Campylobacter jejuni* (*n* = 4). In 2017, four outbreaks were recorded, the highest since systematic data collection on outbreaks began in 1992 (Tables 1 and 2). In addition, two incidents of IID (*Listeria monocytogenes*, *n* = 1; *Salmonella* Dublin, *n* = 1) were investigated which each involved one case, with epidemiological and microbiological links to RDM, but were not designated as outbreaks under the definition (Directive 2003/99/EC). Where data were available, additional details on the number and ages of children affected are provided in Table 2. In total 18 (32.1%) of 54 laboratory confirmed cases were in children aged under 16 years. RDM is not recommended for consumption by children.

**Table 2.** Outbreaks/incidents associated with RDM in England and Wales, 01/01/2014 to 20/12/2017^a^

Year of outbreak	Region	Agent	Vehicle description	Total affected	Laboratory confirmed	Hospitalised	Number of deaths	Setting	Age and gender	Evidence ^b^	Comment (data source^c^)
2014	South West England	STEC O157 PT21/28	Raw cows drinking milk	9	9	2	0	Farm	Seven cases children, two cases adult. Median age of children 5 years; age range of cases 1–49 years	Microbiological and descriptive epidemiological	Seven primary and two secondary cases linked microbiologically and epidemiologically to consumption of raw cows' drinking milk from a single farm. One case was also infected with an identical strain of Salmonella Mbandaka isolated from a sample taken from the bulk tank at the farm. An identical strain of *E. coli* O157 (PT21/28) was identified from the pooled animal faeces collected on the farm premises. All isolates from humans, food and animals were identical on WGS.
2016	National	STEC O157 PT 21/28 stx2	Raw milk cheese	4	4	U	U	Multiple	All adult (age range 31–73), three female and one male	Analytical epidemiological	There were only four cases reported in England, with the remainder (~22) being cases reported from Scotland. All cases were linked by WGS and 71% of primary cases had confirmed exposure to a single artisanal blue cheese product over a short time period.
2016	North West England	*C. jejuni*	Raw cows drinking milk	69	16	0	0	Farm	Mean age of cases was 44 years (range 1–74); 61.9% male. Two children with WGS results; one child aged 8; one child aged 13	Microbiological and analytical epidemiological	Microbiological: WGS identified nine identical *C. jejuni* isolates, seven from human faeces and two from raw milk samples.
2016	South West England	*L. monocytogenes*	Raw milk cheese	1	1	1	1^d^	Farm shop	One adult	Microbiological and epidemiological	This incident was not reported as an outbreak due to only one individual being affected. WGS identified identical isolates in cheese and the human.
2017	South East England	STEC O157 PT21/28 stx2	Raw cows drinking milk	7	7	3	0	Farm	Five cases children. Median age of children 5 years; age range of children 1–11 years. One child aged 1 year; two children aged 5 years; one child aged 10 years; one child aged 11 years.	Microbiological and descriptive epidemiological	Microbiological evidence: Case, food and animal isolates all identical on WGS.
2017	South West England	*Campylobacter* spp.	Raw cows drinking milk	5	5	0	0	Farm	Male × 4, female × 1, age between 41–69 years	Descriptive epidemiological	Five confirmed cases linked epidemiologically to the consumption of raw cows' drinking milk at a farm. From May 2017 until the outbreak, the farm sold raw and pasteurised milk from vending machines at the farm gate. Contamination of the milk with *Campylobacter* was suspected based on the epidemiological evidence but was not confirmed microbiologically.
2017	North West England	*Campylobacter* spp.	Raw cows drinking milk	4	4	0	0	Farm	Male × 2, female × 2, ages between 2–69 years. One child aged 2 years.	Microbiological and descriptive epidemiological	Microbiological evidence: Case and milk isolates all identical on WGS.
2017	Wales	*Campylobacter* spp.	Raw cows drinking milk	18	9	U	0	Farm	Seven cases were aged under 16 (aged 5–13)	Microbiological and descriptive epidemiological	(Personal communication – Public Health Wales)
2017	North West England	*Salmonella* Dublin	Raw cows drinking milk	1	1	U	0	Farm	One child aged <1 year	Microbiological and descriptive epidemiological	**This incident** was **not reported as an outbreak due to only one individual being affected. Isolates of *S*. Dublin detected in bulk milk and farm environmental samples and a clinical case** were **identical on WGS.**

a A Food-borne outbreak is defined ‘an incidence, observed under given circumstances, of two or more human cases of the same disease and/or infection, or a situation in which the observed number of human cases exceeds the expected number and where the cases are linked, or are probably linked, to the same food source’ (Directive 2003/99/EC).

b U, unknown.

c Identical on WGS, refers to isolates that fall within 0 and five single nucleotide polymorphisms of each other.

d Probable cause of death.

During the time frame of this study, there were 12 outbreaks associated with pasteurised milk, including 10 caused by pasteurisation failures (*Campylobacter* species *n* = 3; Salmonella species *n* = 3; STEC O157:H7 *n* = 3; Cryptosporidium species *n* = 1), and two (both Campylobacter species) caused by post-pasteurisation contamination of the milk. The last milk borne outbreak linked to pasteurisation failure occurred in 2011 [7].

In England, national enhanced surveillance systems exist for STEC and *Listeria* and collect detailed, standardised exposure information using an enhanced surveillance questionnaire (ESQ) on every case, which can be used to observe risk factors over time [8, https://www.gov.uk/government/publications/listeria-enhanced-surveillance-questionnaire]. Both ESQs include a question relating to the consumption of RDM, in the exposure period. Analysis of the ESQ's for cases reported between 1st May 2015 and 20th December 2017, identified 19/1284 (1.48%) sporadic cases of STEC (cases related to outbreaks were excluded) and 13/535 (2.43%) sporadic cases of listeriosis reported exposure to RDM. It is not possible to use ESQ data to provide a measure of risk associated with the consumption of RDM, or products made using raw milk, for sporadic cases, because cases are often exposed to more than one potential risk factor for infection, and it is not possible to confirm that consumption of RDM caused the symptoms. However, this analysis does give some indication of the potential of RDM as a vehicle of transmission. Self-reporting may lead to an underestimate; parents may withhold information on their family's consumption of RDM as it is contraindicated for consumption by children. For other pathogens, including *Campylobacter* and non-typhoidal *Salmonella*, collection of exposure data is subject to local variation, the information is not gathered into a central enhanced surveillance database, and it is not possible to assess RDM exposures amongst cases.

Since 2015, the use of routine whole genome sequencing (WGS) at PHE has provided unprecedented sensitivity and accuracy in identifying microbiologically linked cases of infection [9, 10]. However, all RDM-associated STEC O157:H7 outbreaks reported to date have been detected through epidemiological links established by local health protection teams prior to the availability of the WGS results [9, 10]. At the time of the STEC O157:H7 RDM outbreak in 2014, human and RDM isolates were linked using multilocus variable number tandaem repeat analysis, although WGS was used retrospectively to definitively link cases to the outbreak strain [9]. WGS was also used for case ascertainment in the 2016 and 2017 outbreaks, and for definitively linking RDM to clinical cases in the 2017 outbreak [10].

In the UK, reducing foodborne IID has been a key target in the Food Standard's Agency's (FSA) strategy on foodborne disease since its inception in 2000. In July 2015, controls governing the sale and marketing of RDM were reviewed by the FSA and at that time no changes were recommended to the existing control measures (http://www.food.gov.uk/sites/default/files/multimedia/pdfs/board/boardpapers2014/fsa-140704.pdf). Existing control measures include microbiological sampling and testing, appropriate labelling and restrictions on the sale and marketing of RDM. The restrictions on the sale of RDM are governed by the Food Hygiene (England) Regulations (2006) (http://www.legislation.gov.uk/uksi/2006/14/pdfs/uksi_20060014_en.pdf) which includes a microbiological standard of plate count at 30 °C ⩽20 000 cfu/ml and coliforms at <100 cfu/ml. However, a recent assessment of the microbiological quality and safety of RDM on the retail sale in England between 2014 and 2016, found that pathogens and/or indicators of poor hygiene were present in almost half of samples examined [11]. These results demonstrate the importance of continued monitoring and maintaining strict controls on the production and sale of this product.

The data presented within this review indicate that the risk of IID from RDM has likely increased over the last decade, with an increase in the number of outbreaks and incidents associated with RDM since 2014, following an eleven-year period where no RDM outbreaks were reported. Despite the labelling requirements and recommendations that children should not consume RDM, almost a third of outbreak cases were children. Alongside this, there has been an increase in consumer popularity and in registered RDM producers in the UK. In January 2018, there were 165 sites registered for the production of RDM for public consumption, compared to April 2014 when there were 107 registered RDM producers (ref – https://acmsf.food.gov.uk/sites/default/files/acm_1269_revised_final.pdf).

At the Advisory Committee on the Microbiological Safety of Food (ACMSF) (https://acmsf.food.gov.uk/committee/acmsf/acmsfmeets/acmsfmeets/acmsf-meeting-10-may-2018) meeting in May 2018, it was recognised that the microbiological risk associated with consumption of RDM in the UK had increased, reflecting greater levels of exposure due to the increased number of registered producers and volume of consumption, alongside and an increase in the number of outbreaks in human illness associated with RDM. The FSA has subsequently set out additional recommendations to enhance existing controls around registration and hygiene of RDM producers and as of February 2019, is undertaking a timely consultation on the controls (https://www.food.gov.uk/business-guidance/raw-cows-drinking-milk) and provide advice on RDM to consumers (https://www.food.gov.uk/safety-hygiene/raw-drinking-milk). The FSA advises that raw or unpasteurised milk and cream may contain harmful bacteria that cause food poisoning. Pregnant women, infants and small children, elderly people and people with a compromised immune system such as cancer patients, are particularly vulnerable to food poisoning and should not consume raw milk. Severe symptoms of gastrointestinal disease are seen more frequently in younger children, and there is a higher risk of children developing HUS following infection with STEC [8].

In England and Wales, outbreaks linked to the consumption of RDM are rare. The recent increased occurrence, albeit in low frequency, of RDM-related IID incidents is noteworthy, and of concern, and emphasises the continued role of RDM as a cause for human illness. The decision to consume RDM should be informed by the scientific, evidenced-based assessment of the associated risks to public health.
